# Identification and phenotypic characterization of patients with LADA in a population of southeast Mexico

**DOI:** 10.1038/s41598-023-34171-2

**Published:** 2023-04-29

**Authors:** Germán Alberto Nolasco-Rosales, Dania Ramírez-González, Ester Rodríguez-Sánchez, Ángela Ávila-Fernandez, Guillermo Efrén Villar-Juarez, Thelma Beatriz González-Castro, Carlos Alfonso Tovilla-Zárate, Crystell Guadalupe Guzmán-Priego, Alma Delia Genis-Mendoza, Jorge Luis Ble-Castillo, Alejandro Marín-Medina, Isela Esther Juárez-Rojop

**Affiliations:** 1 División Académica de Ciencias de la Salud, Universidad Juarez Autónoma de Tabasco, Villahermosa, Tabasco México; 2 Hospital Juárez de México, Mexico City, México; 3 Hospital Regional de Alta Especialidad “Gustavo A. Rovirosa Pérez”, Villahermosa, Tabasco México; 4 Escuela de Medicina, Universidad Anáhuac Querétaro, Querétaro, Querétaro México; 5 División Académica Multidisciplinaria de Jalpa de Méndez, Universidad Juarez Autónoma de Tabasco, Jalpa de Méndez, Tabasco México; 6 División Académica Multidisciplinaria de Comalcalco, Universidad Juarez Autónoma de Tabasco, Comalcalco, Tabasco México; 7 Departamento de Genética Psiquiátrica, Instituto Nacional de Medicina Genómica, Mexico City, México; 8 Universidad de Guadalajara, CUCS, Guadalajara, Jalisco México

**Keywords:** Biochemistry, Biomarkers, Endocrinology, Medical research

## Abstract

Latent autoimmune diabetes in adults (LADA) has clinical and metabolic features of type 1 and type 2 diabetes. LADA does not have specific features for its diagnosis apart from autoantibody detection; however, these tests are not affordable in clinical settings*.* In this cross-sectional study, we analyzed clinical criteria, metabolic control, pharmacological treatment, and diabetic complications in two groups of patients with diabetes -LADA and T2D- in order to identify specific characteristic of these clinical entities. Finally, we evaluated if the estimated glucose disposal rate (eGDR) and age at diagnosis of diabetes could be used as a diagnostic criterion for LADA. Demographic, biochemical, clinical and treatment were measured in 377 individuals with diabetes. The diagnostics of LADA were determined using Glutamic acid decarboxylase autoantibodies levels. Chi-square test or t-Student test were used to establish differences between groups. To identify factors associated with LADA, a logistic regression analysis was used. Finally, a ROC curve was plotted to assess the possible variables as diagnostic criteria for LADA. The 377 patients with diabetes were separated into 59 patients with LADA and 318 patients with T2D. Patients with LADA showed lower fasting glucose values, fewer diabetic complications, younger age at diagnosis of diabetes, higher insulin use, and higher eGDR in comparison to patients with T2D. Both groups had a mean BMI classified as overweight. The ROC evaluated the sensitivity and specificity, this analysis indicated that an age younger than 40.5 years and an eGDR value higher than 9.75 mg/kg/min correlated better with LADA. These parameters could be useful to identify patients suspected to have LADA at the first level of medical care in the population of southeastern Mexico and refer them to a second level of care.

## Introduction

Individuals with latent autoimmune diabetes in adults (LADA) present autoantibodies that indicate autoimmune pathogenesis. The LADA autoimmune process is milder than type 1 diabetes (T1D); therefore, LADA has a slower progression of beta-cell failure^1^. The majority of individuals with LADA are positive for single islet autoantibodies, while the appearance of multiple islet autoantibodies is a characteristic of T1D^2^. Other differences between LADA and T1D are a later mean age of onset in LADA, a slower rate of beta-cell loss, and a more extended period of insulin independence^3^. In comparison to type 2 diabetes (T2D), individuals with LADA have lower insulin secretion and a faster progression to insulin dependency; furthermore, islet autoantibodies are biomarkers of autoimmune beta-cell destruction that distinguish LADA from T2D^1^.

Various studies have found LADA features halfway T1D and T2D. Individuals with LADA are younger at diagnosis than T2D, but older than those with T1D^4,5^. Individuals with LADA have a lower risk of complications than T2D, however, the risk becomes higher in LADA when there is poor glycemic control^6,7^. Insulin secretion is higher in LADA than in T1D, but lower than in T2D^8^. A hallmark of T2D is insulin resistance; nonetheless, insulin resistance is also present in LADA but is much lower^9,10^. Studies comparing insulin resistance in LADA and T2D have used the HOMA-IR index. As serum insulin collection is not always affordable in clinical contexts, the estimated glucose disposal rate (eGDR) is used instead as a validated score to assess insulin resistance^11^. The parameters of eGDR include easily accessible clinical measures: waist circumference, hypertension, and glycated hemoglobin (HbA1c)^12^. Hence, calculating eGDR in patients with LADA could offer an accessible measure of insulin resistance and the possibility to differentiate them from patients with T2D.

Nowadays, the diagnostic criteria for LADA include: age > 30 years at diagnosis, absence of insulin requirement for at least six months after diagnosis, and the presence of islet autoantibodies^6^. Although LADA has clinical and metabolic features similar to T1D and T2D, it has no categorically defined features^6^. Glutamic acid decarboxylase autoantibodies (GADA) are the most prevalent islet autoantibodies in individuals with LADA^4,13,14^. Therefore, GADA detection is considered sufficient to differentiate between patients with LADA and those with T2D^1^. Nevertheless, autoantibody detection is not affordable in clinical settings, meaning that many clinicians cannot make a confident LADA diagnosis^6^. This uncertainty affects screening strategies and therapeutic approaches; hence, patients with LADA are often misdiagnosed and show worse glycemic control than those with T2D^3^.

In this study we determined the presence of GADA in the serum of individuals diagnosed with T2D who attend a hospital in southeast Mexico. Afterwards, we identified the clinical criteria, metabolic control, drug treatment, and diabetic complications; then, they were separated into patients with LADA and patients withT2DM. Finally, we evaluated whether the estimated glucose clearance rate (eGDR), and the age of diabetes diagnoses could be used as diagnostic criteria for LADA.

## Results

We first selected 566 individuals. We included 377 patients that fulfilled the inclusion criteria. The mean age in our sample was 57.1 ± 10.4 years; 261 individuals were females (69.2%) and 116 were males (30.8%). We found GADA positivity in 15.6% (n = 59) of individuals; these patients were considered as LADA, given they had already accomplished the other two diagnosis criteria. GADA-negative patients were 84.4% (n = 318) and were classified as T2D. We found differences in demographic features between our two groups; for instance, individuals with LADA were younger (53.9 ± 9.3 years) than those with T2D (57.7 ± 10.5). Patients with LADA had 8.6 ± 3.9 years of education, and subjects with T2D had fewer years of education (6.3 ± 4.1) (Table 1).

**Table 1:** Demographic, clinical, anthropometric, biochemical characteristics of patients with LADA and T2D.

Variables	LADA (n = 59)	T2D (n = 318)	χ^2^, t	p
Age (years)	**53.9 ± 9.3**	**57.7 ± 10.5**	**− 2.6**	** < 0.01**
Education (years)	**8.6 ± 3.9**	**6.3 ± 4.1**	**4.0**	** < 0.01**
Age at diagnosis of diabetes	**40.0 ± 7.6**	**44.2 ± 9.5**	**− 3.7**	** < 0.01**
Duration of diabetes (years)	13.7 ± 7.2	13.6 ± 8.4	0.1	0.89
Clinical				
NAFLD/NASH	**2 (3.4%)**	**38 (11.9%)**	**3.9**	**0.03**
DKD	**4 (6.8%)**	**78 (24.6%)**	**9.3**	** < 0.01**
Neuropathy	**11 (18.6%)**	**143 (45.1%)**	**14.4**	** < 0.01**
Cardiovascular complications	**9 (15.3%)**	**135 (42.9%)**	**16.1**	** < 0.01**
Hypertension	27 (45.8%)	170 (53.5%)	1.2	0.17
Alcohol use	8 (13.6%)	50 (15.7%)	0.2	0.42
Tobacco use	7 (11.9%)	18 (5.7%)	3.1	0.08
Anthropometric				
Body mass index (kg/m^2^)	29.4 ± 4.8	29.2 ± 6.2	0.2	0.81
Waist (centimeters)	96.0 ± 9.6	98.7 ± 13.1	− 1.3	0.19
Hip (centimeters)	103.5 ± 10.1	103.2 ± 13.0	0.2	0.87
Systolic (mmHg)	130.9 ± 29.3	125.8 ± 18.2	1.2	0.23
Diastolic (mmHg)	77.8 ± 14.2	75.9 ± 9.5	1.2	0.22
Mean arterial pressure (mmHg)	87.4 ± 31.9	92.6 ± 10.8	− 1.2	0.22
Biochemical				
Fasting glucose (mg/dL)	**150.1 ± 61.9**	**178.4 ± 76.1**	**− 2.4**	**0.02**
HbA1c (%)	8.0 ± 2.4	8.3 ± 2.2	− 0.7	0.49
Total cholesterol (mg/dL)	198.0 ± 48.6	199.9 ± 61.1	− 0.2	0.86
HDL cholesterol (mg/dL)	43.6 ± 6.4	47.3 ± 14.1	− 0.8	0.42
LDL cholesterol (mg/dL)	125.1 ± 44.8	153.5 ± 52.6	− 1.3	0.22
Triglycerides (mg/dL)	180.0 ± 107.6	202.2 ± 112.5	− 1.1	0.26
eGDR	**8.5 ± 4.6**	**5.9 ± 2.5**	**3.3**	** < 0.01**
GADA (IU/mL)	**18.9 ± 16.8**	**2.4 ± 1.1**	**7.4**	** < 0.01**

Significant values are in [bold].

LADA, Latent autoimmune diabetes in adults; T2D, Type 2 diabetes; NAFLD, Nonalcoholic fatty liver disease; NASH, Nonalcoholic steatohepatitis; DKD, Diabetic kidney disease, eGDR, Estimated glucose disposal rate; GADA, Glutamic acid decarboxylase antibodies. We used the Chi-squared test for categorical variables, and the t-Student test for numerical data. A p-value < 0.05 was considered significant.

Some clinical features were also different between LADA and T2D: at diagnosis of diabetes, individuals with LADA were younger than those with T2D (40.0 ± 7.6 vs. 44.2 ± 9.5 years). Regarding diabetes complications, we found lower frequencies of complications in patients with LADA. NAFLD, NASH, DKD, neuropathy, and cardiovascular complications were most common in patients with T2D. Other clinical features such as alcohol and tobacco use were equally frequent in LADA and T2D (Table 1).

Both groups, LADA and T2D had similar anthropometric characteristics. The mean BMI was classified as overweight (29.4 and 29.2 kg/m^2^). Individuals with T2D showed higher levels of fasting glucose. Other biochemical parameters were similar in LADA and T2D groups (Table 1). For instance, eGDR was greater in patients with LADA (8.5 ± 4.6) than those with T2D; furthermore, the mean eGDR in patients with T2D (5.9 ± 2.5) was below the insulin resistance threshold (< 8.0) GADA levels in patients with LADA were 18.9 ± 16.8 IU/mL (Table 1). The majority of patients with LADA (n = 46) presented GADA levels between 5 and 20 IU/mL.

The pharmacologic treatment presented some differences between patients with LADA and T2D. Individuals with LADA used insulin more frequently than those with T2D (79.7% vs. 62.6%, respectively). Short-acting insulin use was less frequent in those with LADA (25.5% vs. 44.7%), while long-acting insulin use was more frequent (70.2% vs. 51.3%). Regarding antidiabetic drugs, metformin was the most frequently used in both groups. Metformin was predominant in LADA (96.6% vs. 84.6%). On the other hand, a large number of individuals with T2D have being used linagliptin (27.7% vs. 11.9%). Doses of pharmacological treatment were similar between patients with LADA and T2D. Nevertheless, short-acting insulin doses were lower in patients with LADA (15.0 ± 6.2 vs. 20.2 ± 14.6 mg) (Table 2).

**Table 2:** Diabetes therapy in patients with LADA and T2D.

Treatment	LADA		T2D		χ^2^, p	t, p
	n = 59, %	Dose	n = 318, %	Dose		
Insulin	**47 (79.7%)**	37.3 ± 18.7 IU/day	**199 (62.6%)**	39.8 ± 23.7 IU/day	**6.4, < 0.01**	− 0.7, 0.5
Short-Acting insulin	**12 (25.5%)**	**15.0 ± 6.2** IU/day	**89 (44.7%)**	**20.2 ± 14.6** IU/day	**5.8, 0.01**	**− 2.2, 0.03**
Intermediate-Acting insulin	18 (38.3%)	35.3 ± 11.4 IU/day	80 (40.2%)	35.0 ± 14.7 IU/day	0.1, 0.47	0.1, 0.94
Long-Acting insulin	**33 (70.2%)**	28.4 ± 9.8 IU/day	**102 (51.3%)**	26.4 ± 11.7 IU/day	**5.5, 0.01**	0.9, 0.36
Metformin	**57 (96.6%)**	1828.1 ± 541.2 mg/day	**269 (84.6%)**	1770.4 ± 564.3 mg/day	**6.2, < 0.01**	0.7, 0.48
Glibenclamide	28 (47.5%)	9.6 ± 3.3 mg/day	134 (42.1%)	11.0 ± 4.1 mg/day	0.6, 0.27	− 1.7, 0.09
Linagliptin	**7 (11.9%)**	5.7 ± 1.9 mg/day	**88 (27.7%)**	5.4 ± 1.6 mg/day	**6.6, < 0.01**	0.5, 0.61

Significant values are in [bold].

LADA, Latent autoimmune diabetes in adults; T2D, Type 2 diabetes. We used Chi-squared test for categorical variables, and t-Student test for numerical data. A p-value < 0.05 was considered as significant.

Logistic regression included all significant variables associated with the univariable analysis. We found that being younger (OR: 1.1, 95% CI: 1.0–1.1), having more years of education (OR: 1.2, 95% CI: 1.1–1.3), using insulin (OR: 4.9, 95% CI: 1.2–20.1), and having higher eGDR (OR: 1.6, 95% CI: 1.3–1.9) were more frequent in individuals with LADA. Conversely, older age at diagnosis of diabetes (OR: 0.9, 95% CI: 0.9–1.0), DKD (OR: 0.2, 95% CI: 0 -0.6), neuropathy (OR: 0.3, 95% CI: 0.1–0.8), and linagliptin use (OR: 0.1, 95% CI: 0.0–0.3) were more frequent in patients with T2D (Table 3).

**Table 3:** Logistic regression analysis of factors associated in patients with LADA.

Variable	OR	95% CI	p
Age (years)	1.07	1.00–1.14	**0.04**
Education (years)	1.19	1.06–1.34	** < 0.01**
Diagnosis of diabetes (age)	0.92	0.85–0.99	**0.02**
DKD	0.15	0.04–0.62	** < 0.01**
Neuropathy	0.32	0.13–0.78	**0.01**
Insulin use	4.99	1.24–20.08	**0.02**
Linagliptin use	0.06	0.01–0.29	** < 0.01**
eGDR	1.57	1.28–1.93	** < 0.01**

Significant values are in [bold].

LADA, Latent autoimmune diabetes in adults; T2D, Type 2 diabetes; DKD, Diabetic kidney disease; eGDR, Estimated glucose disposal rate. We used binary logistic regression. A p-value < 0.05 was considered significant.

**Figure 1:**
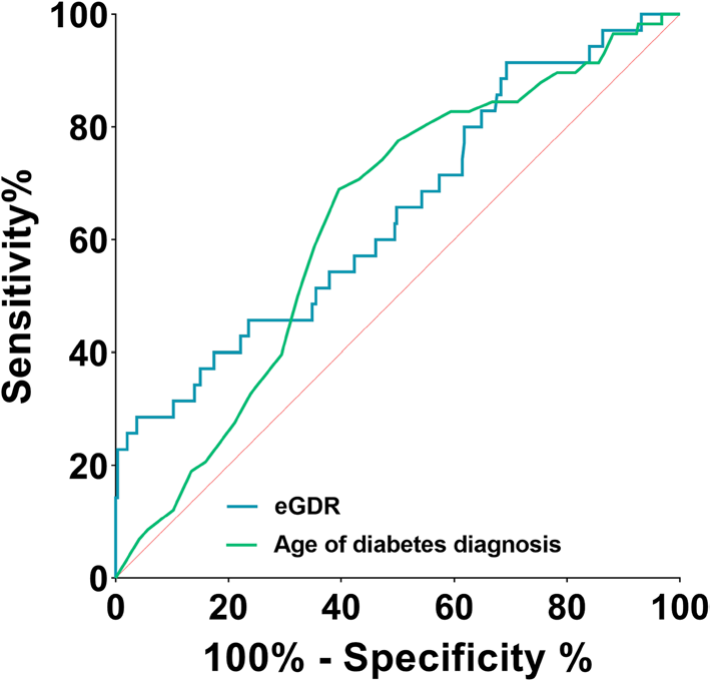
The receiver operating (ROC) curve of factors associated with LADA. eGDR, Estimated glucose disposal rate. The area under curve for eGDR and age of diabetes diagnosis are 0.674 and 0.632, respectively.

We performed a ROC curve analysis to evaluate the sensitivity and specificity of being in the LADA group in our sample; we used the significant variables from the logistic regression (Fig. 1). The area under the curve (AUC) for age at diagnosis of diabetes and eGDR were 0.6 (95% CI: 0.6–0.7, p: 0.001) and 0.7 (95% CI: 0.6–0.8, p: 0.004) respectively. In Table 4, we show the Youden index, which indicates the age at diagnosis younger than 41 years and eGDR greater than 9.75 correlated better with our group with LADA. Besides, positive and negative predictive values showed that both criteria are better for discarding than for diagnosis confirmation (Table 4).

**Table 4:** Sensitivity and specificity of age at diabetes diagnosis and eGDR cut-off points.

Age at diabetes diagnosis							
Cut-off	Sensitivity	Specificity	Youden Index	False positive rate	False negative rate	Positive predictive value	Negative predictive value
< 31	0.07	0.96	0.03	0.04	0.93	0.24	0.85
< 36	0.28	0.79	0.07	0.21	0.72	0.20	0.86
** < 41**	**0.69**	**0.60**	**0.29**	**0.40**	**0.31**	**0.24**	**0.91**
< 46	0.83	0.41	0.23	0.59	0.17	0.21	0.93
< 51	0.90	0.22	0.11	0.78	0.10	0.18	0.92
< 56	0.97	0.12	0.08	0.88	0.03	0.17	0.95
< 61	0.98	0.07	0.06	0.93	0.02	0.16	0.96
< 66	1.00	0.03	0.03	0.97	0.00	0.16	1.00

eGDR							
Cut-off	Sensitivity	Specificity	Youden Index	False positive rate	False negative rate	Positive predictive value	Negative predictive value
> 4	0.91	0.24	0.15	0.76	0.09	0.13	0.96
> 5	0.80	0.37	0.17	0.63	0.20	0.13	0.94
> 6	0.60	0.51	0.11	0.49	0.40	0.13	0.91
> 7	0.51	0.62	0.14	0.38	0.49	0.14	0.92
> 8	0.43	0.78	0.20	0.22	0.57	0.19	0.92
> 9	0.31	0.88	0.20	0.12	0.69	0.24	0.89
> **9.75**	**0.29**	**0.96**	**0.25**	**0.04**	**0.71**	**0.48**	**0.92**
> 10	0.26	0.97	0.23	0.03	0.74	0.53	0.92

Significant values are in [bold].

eGDR, Estimated glucose disposal rate. Age at diagnosis lower than 41 years and an eGDR higher than 9.75 correlates better with LADA and have the best positive and negative predictive value.

## Discussion

In our sample, patients with LADA had similar features to patients with T2D; nonetheless, they had fewer diabetic complications, they were younger, had a higher use of insulin and higher eGDR when compared to individuals with T2D. We suggest that this discrepancy can be partially attributed to the epidemiological characteristics of the population of southeast Mexico. Additionally, the clinical characteristics of individuals with T2D indicated poor glycemic control, obesity (BMI) and hypertension.

In a multicenter ADOPT study, it was found that participants with LADA and T2D were similar in terms of overweight/obesity; nevertheless, those with LADA were more insulin resistant and tended to be obese, but leaner than individuals with T2D^9^. Brahmkshatriya et al.^8^ used a different age criterion (> 25 years) for LADA and found that these individuals had a slim phenotype; interestingly, their study suggested that a younger age of LADA onset is associated with a slim phenotype. In contrast, we observed that individuals with LADA had a phenotype similar to T2D. On the other hand, a Swedish study found that patients with LADA and a healthy lifestyle showed higher levels of GADA and worse beta-cell function (HOMA-B) than those with a poor lifestyle; however, they presented less insulin resistance (HOMA-IR). One explanation could be that people with a poor lifestyle are overweight/obese and insulin resistant: two factors that increase the demand on the beta cells, which could unmask an autoimmune process at an earlier stage^10^.

Regarding diabetes complications, our patients with LADA showed lower frequencies of NAFLD/NASH, DKD, neuropathy and cardiovascular complications. Similarly, other researchers have found that a poor glycemic control increases the risk of microvascular complications in individuals with LADA^3^. Likewise, other studies have demonstrated the role of poor glycemic control in complications of prolonged diabetes^1,15^.

In our population, a mean eGDR of 8.51 ± 4.55 mg/Kg/min was found in patients with LADA, which was higher than in individuals with T2D. This difference may be because this parameter depends on HbA1c, waist circumference, and hypertension values, for its determination. It is known that these factors depend on the interaction of environmental, behavioral (diet, lifestyle) and genetic factors inherent to our Mexican population (presence of genetic variants that predispose to metabolic disorders, hypertension)^16^. Similarly, Zinman et al.^17^ found in patients with LADA a lower degree of insulin resistance and a lower probability of metabolic syndrome. Some authors propose that insulin resistance participates in the promotion of autoimmune diabetes due to the increase in insulin demand, and this can accelerate the appearance of the disease in people with an autoimmune process. In mild autoimmunity, the participation of factors related to insulin resistance in the progression to diabetes is considered^9^.

Low eGDR is a marker associated with insulin resistance. In this sense, a study of patients with T1D, showed that those who presented an eGDR lower than 6.6 mg/Kg/min had a higher probability of presenting acanthosis nigricans^18^. Some reports suggest that the prevention of insulin resistance would reduce atherosclerotic disease up to 40%. Zabala, et al.^19^ found that a low eGDR is associated with cerebrovascular events and a higher probability of death^19^. Therefore, eGDR can be used as a marker to assess insulin resistance and the risk of cardiovascular disease^20^.

Nowadays, the consideration of specific criteria is suggested when suspecting LADA, including: age > 30 years at diagnosis and the absence of insulin requirement for at least 6 months after diagnosis^6^. Additionally, clinical characteristics and the presence of autoantibodies are frequently used in studies to define LADA cases. Carlsson for instance, considered that for research purposes, it is enough to measure GADA to distinguish LADA from T2D^1^. Studies carried out in Chinese and European populations showed that GADA were the most frequent autoantibodies in patients with LADA^4,14^. Various authors suggest that other criteria (BMI, patient age, fasting blood glucose, glycosylated hemoglobin) should also be used for the diagnosis of LADA, and be adjusted according to the population studied^6,21^. However, GADA positivity rates vary widely among reports depending on the population sampled and the screening methodology. We found 18.55% of GADA-positive patients in our sample, a high detection rate when compared to studies performed in other populations (1.85–14%)^22,23^. On the other hand, these data are similar to that reported in a Spanish population (27%)^24^; likewise, Cardoso et al.^25^ study had a relatively high antibody detection rate (32.26%) in Mexican patients.

It has been reported that GADA levels can fluctuate or even become negative during the disease course, but patients with higher levels of GADA tend to remain positive and have an accelerated decline of the beta-cell function^26,27^. Due to the varied antibody titers reported in different populations, the criteria for the diagnosis of LADA are under constant discussion^28^.

Our findings indicated that individuals with LADA belong to a subtype of patients with a T2D phenotype (a high BMI; overweight), but with lower fasting glucose levels and a higher eGDR than those observed in T2D. The Youden index indicated that a mean age at diagnosis of diabetes of 41 years and a mean eGDR value of 9.75 mg/Kg/min correlated better with LADA. Nonetheless, positive and negative predictive values showed that both features would be better for discarding LADA than for its diagnosis. In this sense, these parameters could be used to identify patients suspected of having LADA at the first level of medical care. Due to the phenotypic heterogeneity observed in LADA between populations, it is necessary to have parameters that allow the identification of patients with a high suspicion of LADA, so that they are appropriately referred to the second level of clinical care.

The present study has limitations to be considered. We only evaluated patients from a single referral center in Southeast Mexico; therefore, other Mexican populations can differ from our data. Also, our sample was taken from a Diabetes Clinic where all patients are on intensive therapies; they were referred from other centers because they did not achieve a glycemic control. Hence, our studied population cannot be considered a typical T2D population. Lastly, we could not establish causal relationships because of the cross-sectional nature of this study.

## Conclusions

We found that patients with clinical criteria for LADA were GADA positive in a population from southeastern Mexico. Patients diagnosed with LADA in our population presented low fasting glucose, fewer complications from the disease, younger age and younger age at onset, higher insulin use and higher eGDR than patients with T2D. Our results indicate that a mean age at diagnosis of 40.5 years and a mean eGDR value of 9.75 mg/kg/min correlate better with LADA. Since GADA screening is unavailable in several clinical centers, these parameters may be useful to identify patients with a high suspicion of LADA at the first level of care in populations of southeastern Mexico, in order to refer them to a second level of care. It is necessary to perform further research on LADA features in the Mexican population since it could differ from the population characteristics in other countries.

## Materials and methods

### Study population

**Figure 2:**
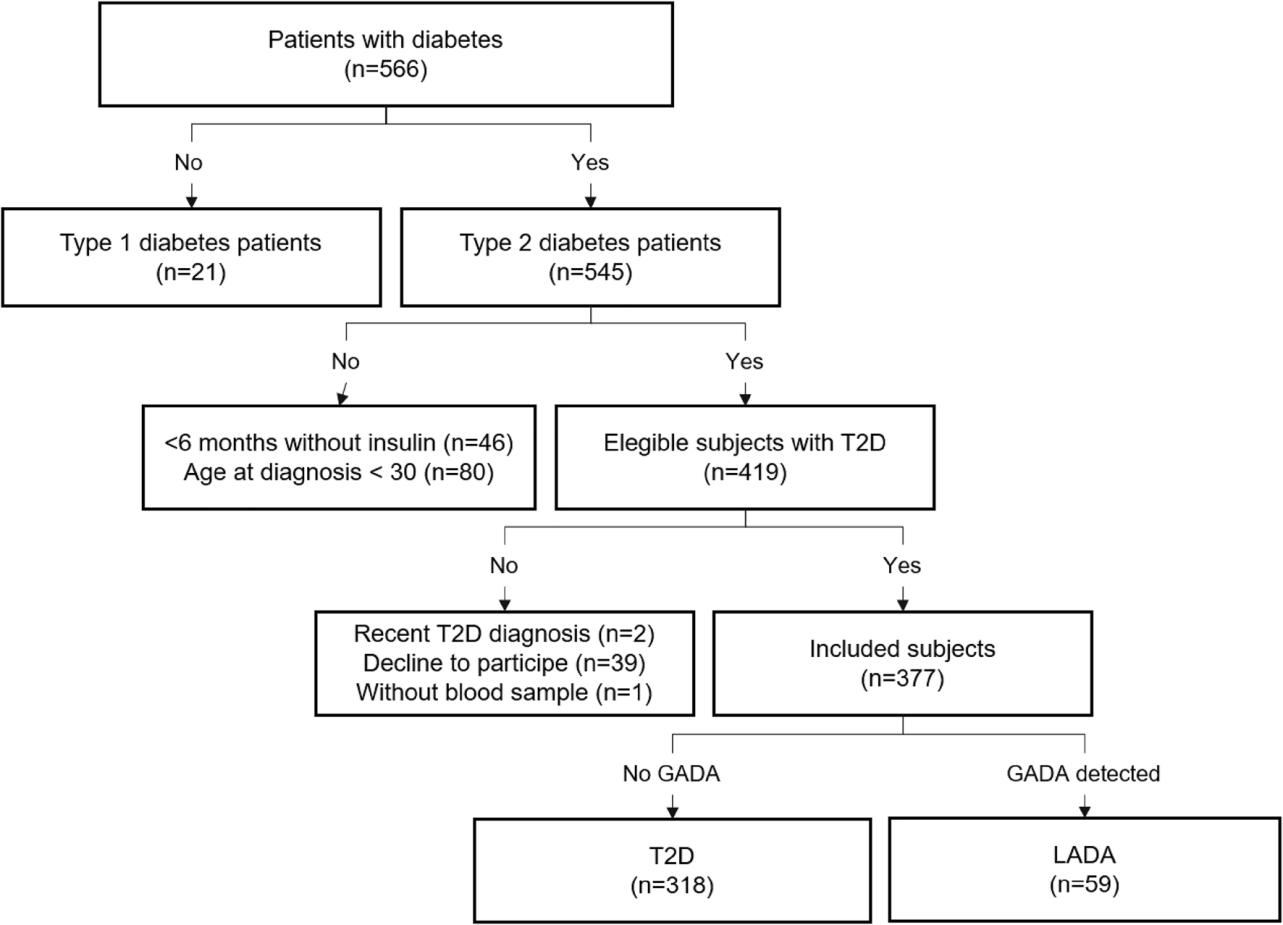
Flow chart for patient selection and classification. GADA, Glutamic acid decarboxylase antibodies; LADA, Latent autoimmune diabetes in adults.

We performed a cross-sectional study. The participants were recruited at the Diabetes Clinic of the High Specialization Regional Hospital “Dr. Gustavo A. Rovirosa Pérez” from January 2020 to May 2021. This Hospital cares for individuals from the Chiapas, Veracruz and Tabasco states (Southeast Mexico). We invited individuals with previous T2D diagnoses to participate in this study. A diabetologist evaluated and diagnosed these patients based on the World Health Organization (WHO) criteria^29^. The inclusion criteria were (1) diagnosis of T2D, (2) older than 30 years of age at diagnosis of diabetes, (4) at least 6 months after diagnosis without insulin therapy requirement, (5) agreeing to participate in the study, and signed an informed consent before the interview. We excluded patients with (1) a diagnosis of T1D, (2) less than a year of diagnosis of T2D, and (3) subjects without a blood sample. A sample of 377 patients completed the inclusion criteria. We determined the GADA levels of all individuals in the sample; we classified them as LADA subjects with present antibodies and T2D patients with absent antibody. The final sample was divided into two groups: 59 patients with LADA and 318 patients with T2D (Fig. 2).

### Measurements sociodemographic and clinical features

We applied a structured questionnaire to obtain sociodemographic data (age, gender, occupation, education, marital status, and socioeconomic status). From medical records, we collected: age at diagnosis, diabetes complications, comorbidities, pharmacotherapy, alcohol consumption and tobacco consumption. The time of living with diabetes was calculated in years, from the time of diagnosis to the interview day. Diabetes complications included non-alcoholic fatty liver, disease/non-alcoholic steatohepatitis (NAFLD/NASH), diabetic kidney disease (DKD), neuropathy, and cardiovascular complications (cardiovascular complications enclosed coronary heart disease, cardiomyopathy, stroke, and diabetic foot syndrome). The comorbidities evaluated were hypertension and dyslipidemia. In terms of insulin use, we classified insulin as short-acting (Lispro), intermediate-acting (NPH, Lispro protamine), and long-acting (glargine, detemir).

### Anthropometric parameters

We measured weight, height, waist circumference, and hip circumference after the interview. We calculated BMI as weight in kilograms divided by the square of height in meters (kg/m^2^). Blood pressure measurements followed the protocol recommended by the Official Mexican Standard and the American Heart Association.

### Biochemical parameters

We drew blood samples from every participant and collected them in tubes with EDTA and no other anticoagulants. Fasting glucose, total cholesterol, HDL cholesterol, LDL cholesterol, and triacylglycerols were determined in blood serum using a Clinical Chemistry System from Random Access Diagnostics. We determined glycated hemoglobin and GADA concentrations by an enzymatic immunoassay method (Human Glycated Hemoglobin A1c ELISA Kit, Human Anti-Glutamic Acid Decarboxylase ELISA Kit; MyBioSource).

### Estimated glucose disposal rate (eGDR)

We calculated the eGDR using the formula: eGDR = 21.158−(0.09 × waist [cm])−(3.407 × hypertension [yes = 1, no = 0])−(0.551 × HbA1c [%])^12^.

### Diabetes classification

We diagnosed patients with LADA based on the following criteria: adult-onset of diabetes (> 30 years of age at diagnosis), absence of insulin requirement for at least six months after diagnosis, and presence of GADA (levels of ≥ 5 IU/mL)^30^. GADA positivity was the main classification factor, as we used age at diagnosis of diabetes and absence of insulin requirement as inclusion criteria for patients with T2D.

### Statistical analysis

Means ± standard deviations describe numerical variables, and frequencies and percentages express categorical data. We compared two groups, one with LADA and another with T2D. We used the Chi-squared test for categorical variables and the t-Student test for numerical data. Significant variables in previous analyses were included as covariables in a binary logistic regression; results were expressed as OR and 95% CI for being in the LADA group. The receiver operating (ROC) curve evaluated the sensitivity and specificity of significant numerical variables in logistic regression. We calculated the Youden index to estimate the best cut-off point for sensitivity and specificity. A p-value < 0.05 was considered significant. All data were analyzed using SPSS v. 26 and GraphPad Prism 9.

### Ethical considerations

We followed the Official Mexican Standard NOM-012-SSA3-2012 guidelines and the ethical principles of the Declaration of Helsinki. The Ethics Committee of High Specialization Regional Hospital “Dr. Gustavo A. Rovirosa Pérez” approved the study (00228/19; December 3rd, 2019). Every patient participated voluntarily, without receiving any remuneration and signed an informed consent. Additionally, they received verbal and written information about the objectives of this study. Participation in this study was not treatment dependent and did not change the medical care that the hospital provides. We registered this study on clinicaltrials.gov on February 11th, 2022, as NCT05235672.

### Statement and ethical considerations of experiments involving human individuals

The Ethics Committee of The High Specialization Regional Hospital “Dr. Gustavo A. Rovirosa Pérez” approved the study (00228/19; December 3rd, 2019).

### Statement of methods

All methods were performed according to the Official Mexican Standard NOM-007-SSA3-2011 for clinical laboratory determinations and the Official Mexican Standard NOM-012-SSA3-2012 for research in human individuals.

### Statement of informed consent

The present study was performed according to the ethical principles of the Declaration of Helsinki. All patients participated voluntarily without receiving any remuneration and signed an informed consent. Additionally, they received verbal and written information about the objectives of this study. The participation in this study was not treatment dependent and did not change the medical care that the hospital provides.

## Supplementary Information

Supplementary Tables.

## Data Availability

The datasets used and analyzed during this study are included in the published article and its supplementary information files.
